# Women's Acceptability of Misoprostol Treatment for Incomplete Abortion by Midwives and Physicians - Secondary Outcome Analysis from a Randomized Controlled Equivalence Trial at District Level in Uganda

**DOI:** 10.1371/journal.pone.0149172

**Published:** 2016-02-12

**Authors:** Amanda Cleeve, Josaphat Byamugisha, Kristina Gemzell-Danielsson, Nazarius Mbona Tumwesigye, Susan Atuhairwe, Elisabeth Faxelid, Marie Klingberg-Allvin

**Affiliations:** 1 Department of Women’s and Children’s Health, Karolinska Institutet, Stockholm, Sweden; 2 WHO Center for Human Reproduction, Karolinska University Hospital, Stockholm, Sweden; 3 Department of Obstetrics and Gynaecology, Mulago Hospital, Kampala, Uganda; 4 Department of Obstetrics and Gynaecology, Makerere University College of Health Sciences, Kampala, Uganda; 5 Department of Epidemiology and Biostatistics, School of Public Health, Makerere University, Kampala, Uganda; 6 Department of Public Health Sciences, Global Health (IHCAR), Karolinska Institutet, Stockholm, Sweden; 7 School of Education, Health and Social Studies, Dalarna University, Falun, Sweden; NHS lothian and University of Edinburgh, UNITED KINGDOM

## Abstract

**Objective:**

This study aimed to assess women´s acceptability of diagnosis and treatment of incomplete abortion with misoprostol by midwives, compared with physicians.

**Methods:**

This was an analysis of secondary outcomes from a multi-centre randomized controlled equivalence trial at district level in Uganda. Women with first trimester incomplete abortion were randomly allocated to clinical assessment and treatment with misoprostol by a physician or a midwife. The randomisation (1:1) was done in blocks of 12 and stratified for health care facility. Acceptability was measured in expectations and satisfaction at a follow up visit 14–28 days following treatment. Analysis of women’s overall acceptability was done using a generalized linear mixed-effects model with an equivalence range of -4% to 4%. The study was not masked. The trial is registered at ClinicalTrials.org, NCT 01844024.

**Results:**

From April 2013 to June 2014, 1108 women were assessed for eligibility of which 1010 were randomized (506 to midwife and 504 to physician). 953 women were successfully followed up and included in the acceptability analysis. 95% (904) of the participants found the treatment satisfactory and overall acceptability was found to be equivalent between the two study groups. Treatment failure, not feeling calm and safe following treatment, experiencing severe abdominal pain or heavy bleeding following treatment, were significantly associated with non-satisfaction. No serious adverse events were recorded.

**Conclusions:**

Treatment of incomplete abortion with misoprostol by midwives and physician was highly, and equally, acceptable to women.

**Trial Registration:**

ClinicalTrials.gov NCT01844024

## Introduction

Complications from unsafe abortions continue to be a major contributor to the global maternal mortality ratio [1]. The majority of abortion related deaths occur in low income-countries with restrictive abortion laws and low contraceptive prevalence [2]. Young and rural women, and women with low socio-economic status, are especially vulnerable to unintended pregnancies and unsafe abortions, demonstrating the inequity in safe abortion care access across the globe [3]. Sub-Saharan Africa has the highest global burden of unsafe abortion and is also where the highest rates of pregnancy related deaths are found [1]. In order to address the burden of unsafe abortion, universal access to post abortion care (PAC), consisting of emergency treatment of complications from spontaneous or induced abortions and contraceptive services, is crucial [4].

In Uganda, abortion complications are a public health issue placing a huge burden on the health care system and societies at large [5]. Although stigmatized and legally restricted, abortions are common and thought to contribute to 26% of the maternal mortality [6]. Health care providers in Uganda are scarce; especially physicians in rural areas are lacking [7], and few midwives are trained in PAC [8]. Task shifting is a process where tasks are delegated to less specialized health care providers. A task shift between midwives and physicians could expand access to care and result in more cost-effective and equitable health care services [9]. Results from the analysis of the primary outcome in this randomized controlled trial showed that midwives can diagnose and treat incomplete abortion with misoprostol as safe and effective as compared with physicians [10].

Misoprostol is proven safe and effective for treatment of incomplete abortion in the first trimester [11–13] and highly suitable in low resource settings as it is cost-effective, resource saving and heat stable [14, 15]. Studies from Sub-Saharan Africa have shown that misoprostol for treatment of incomplete abortion is highly acceptable to women [11, 12, 16–19] and providers find it preferable [17]. Acceptability is an important component of access reflecting the contextual adaptation of services, the patient-provider relationship, and the judged appropriateness of care [20]. However, no randomized controlled trial has previously assessed women´s acceptability of PAC, when diagnosis and treatment is provided by midwives compared with physicians. Increasing access to care through task shifting requires an understanding of women´s acceptability when care is provided by different cadres. This study aimed to assess women´s acceptability of misoprostol treatment for incomplete abortion by midwives compared with physicians at district level in Uganda. Further objectives were to assess how the treatment experience influenced overall acceptability.

## Methods

### Details of ethical approval

The study was approved by the Scientific and ethical review group at the Reproductive Health and Research Department, WHO, Geneva. Ethical approval was further obtained from the Research Ethics Committee, Makerere University, Dnr: 2012–129, Uganda National Council for Science and Technology Dnr: HS 1314 and the Swedish regional ethical review board at Karolinska Institutet Dnr: 2013/2;9.

### Trial design and participants

This is an analysis of secondary outcomes from a multicentre randomized controlled equivalence trial. The trial was designed primarily to compare safety and effectiveness of diagnosis and treatment of incomplete abortion with misoprostol by midwives and physicians (reported elsewhere) [10], and secondly to measure women’s acceptability. The focus of this paper is women’s acceptability of treatment. The study was conducted at district level in six different health care facilities in central Uganda. The health care facilities included three hospitals and three health care centres level IV in rural, peri-urban and urban settings. A health care centre level IV is smaller than a hospital with more basic care provision; it should have a doctor and possibilities to provide emergency obstetric services. Data collection was conducted between April 2013 and July 2014. Following the run-in period there were no changes to the trial design. The study was not masked. The trial and study protocol followed the CONSORT guidelines for non-inferiority and equivalence randomized controlled trials [21]. Inclusion criteria were women with signs of incomplete abortion i.e. contractions, pain and vaginal bleeding during pregnancy, an open cervical os and sometimes partial expulsion of products of conception. Exclusion criteria were; a uterine size of more than 12 weeks of gestation; complete abortion; suspected ectopic pregnancy; unstable hemodynamic status and shock; signs of pelvic infection or sepsis; and a known allergy to misoprostol.

### Intervention and procedure

Physicians and midwives involved in PAC at the different facilities were eligible for participation. None of the providers who were asked to participate declined. The health care providers were trained in PAC according to a standardized five day training programme [22, 23] covering diagnosing and treating incomplete abortion, values clarification, and post abortion contraceptive methods and counselling. Values clarification aims to improve providers’ attitudes towards and knowledge of abortion, and to help providers offer care that shows respect for women’s decisions and reproductive rights [23]. A number of midwives at each facility were trained to be research assistants in the study and were responsible for eligibility screening, enrolment of participants, randomization and follow up visits. Women who were eligible and consented to participation were randomly allocated to a midwife (intervention) or a physician (standard care/control) for diagnosis and treatment. Ultrasound was not systematically used during first or second visit and gestational age was determined by self-reported last menstrual period and bimanual palpation by the provider whom the woman was randomised to. One single dose of 600mcg misoprostol orally was given to all participants [24]. Analgesics (paracetamol and/or ibuprofen most commonly provided) and oral antibiotics were offered according to Ugandan national PAC guidelines. Treatment counselling, provided by the provider to whom the woman was randomised, included information on expected bleeding, pain, and abnormal symptoms following treatment that should prompt care seeking. Before discharge all women were offered contraceptive counselling and provision, and provided with a follow up-date. A reimbursement for travel expenses was offered to all women when returning for the follow up visit. Adverse events were recorded in a separate protocol. Clinical procedures for all women participating in the study followed treatment guidelines according to the WHO and the International Federation of Gynaecology and Obstetrics (FIGO) [24, 25].

### Outcomes

Outcomes were measured by a research assistant at a follow up visit, within 14 to 28 days after the initial visit. Standardized questionnaires were used to collect information about women’s acceptability of the treatment. Acceptability was measured in expectations (as expected/easier than expected/worse than expected) and satisfaction (would recommend the treatment to a friend yes/no). Overall acceptability (satisfactory/non-satisfactory) was regarded as a dependent variable and calculated by merging the two questions measuring acceptability. Satisfactory meant that the treatment was found to be “as expected/easier than expected” and that the woman would recommend the treatment to a friend. Non-satisfactory meant that treatment was experienced as “worse than expected” or the women would not recommend the treatment to a friend. Measures such as bleeding and pain following treatment, feeling calm and safe following treatment, unscheduled visits, treatment outcome (complete/incomplete abortion), adverse events, and side effects, were regarded as independent variables reflecting women’s treatment experience. Bleeding was measured as the intensity of bleeding in relation to normal menstruation (same as/more than/less than), and pain experienced following treatment was measured using a visual analogue scale (VAS) with a 0 to 10 point scale. Socio-demographic background and reproductive history were considered to possibly affect overall acceptability.

### Sample size

The sample size was calculated based on the treatment outcome (complete or incomplete abortion). The calculations were based on the objective of showing two-sided equivalence assuming that the rate of incomplete abortions, for each study group (midwife/physician), could be four per cent. An acceptable completion rate between the two providers was predefined based on clinical significance (related to treatment outcome), and ranged between -4% to 4%. With a power of 80% and two-sided 95% confidence interval (CI), the sample size becomes 452 per arm, in order to establish equivalence. Compensating for a 10% loss to follow up the required total sample size was 994 women.

### Randomisation

The randomisation (1:1) was conducted in blocks of 12 and stratified for health care facility. A computerized random number generator was used to generate a randomization list with codes, each linked to one of the two study groups, from 1 to 994. The randomization list and sequentially numbered, opaque and sealed envelopes, each containing a random allocation, were prepared at the coordinating centre in Mulago Hospital, and later opened by the research assistants after obtaining consent. Both verbal and written informed consent for participation was sought and obtained from all women included in the study. Women consented that data collected for study purposes were entered in anonymised study protocols. Data management was organized at the coordinating centre and data were entered continuously throughout data collection. Protocols were checked for accuracy and corrected by the study coordinator, after consultations with research assistants.

### Statistical analyses

Descriptive statistics were used to present background characteristics and categorical outcomes. The difference in the proportion of subjects with overall acceptability between the provider groups is called the risk difference. The risk difference was estimated using a generalized linear mixed-effects model with group as a fixed effect and health-care facility as random effect. The random effects were estimated using an unstructured variance-covariance structure. 1000 bootstrap simulations were used to estimate the confidence interval for the risk difference. In addition, we estimated an adjusted risk difference where the model was extended with following fixed effects: age (<25 *vs* ≥25 years), marital status (single *vs* married or cohabiting), education (none or primary *vs* secondary or tertiary), number of pregnancies (1 *v*s >1), and parity (0 *vs* ≥1), and reported induced current abortion (yes vs. no). The adjusted risk difference was estimated as the predicted risk difference at the average of all included covariates. Equivalence between the two study groups can be stated if the 95% CI of the risk difference lies completely within the limits of equivalence (–4% to 4%). Univariate logistic regression was used to assess the relationship between the dependent variable (overall acceptability), and the independent variables (socio-demographic background, reproductive history, treatment experience, including treatment outcome). Independent variables found to be statistically significant were then added in a multivariate logistic regression analysis. P-values ≤0.05 were considered statistically significant. Data was entered into EpiData 3.1 and analysed in Stata version 13. Statistical analysis of women’s overall acceptability was analysed using the Ime4 package in R version 3.1.1.

Researchers from the WHO research centre at Karolinska Institutet, Stockholm, and Mulago Hospital/Makerere University, Kampala, developed and coordinated the study. Approval was sought and later approved by the Scientific and ethical review group at the Reproductive Health and Research Department, WHO, Geneva. Ethical approval was obtained from the Research Ethics Committee, Makerere University, Dnr: 2012–129, Uganda National Council for Science and Technology Dnr: HS 1314 and the Swedish regional ethical review board at Karolinska Institutet Dnr: 2013/2;9. The trial is registered at ClinicalTrials.gov NCT 01844024.

## Results

**Fig 1:**
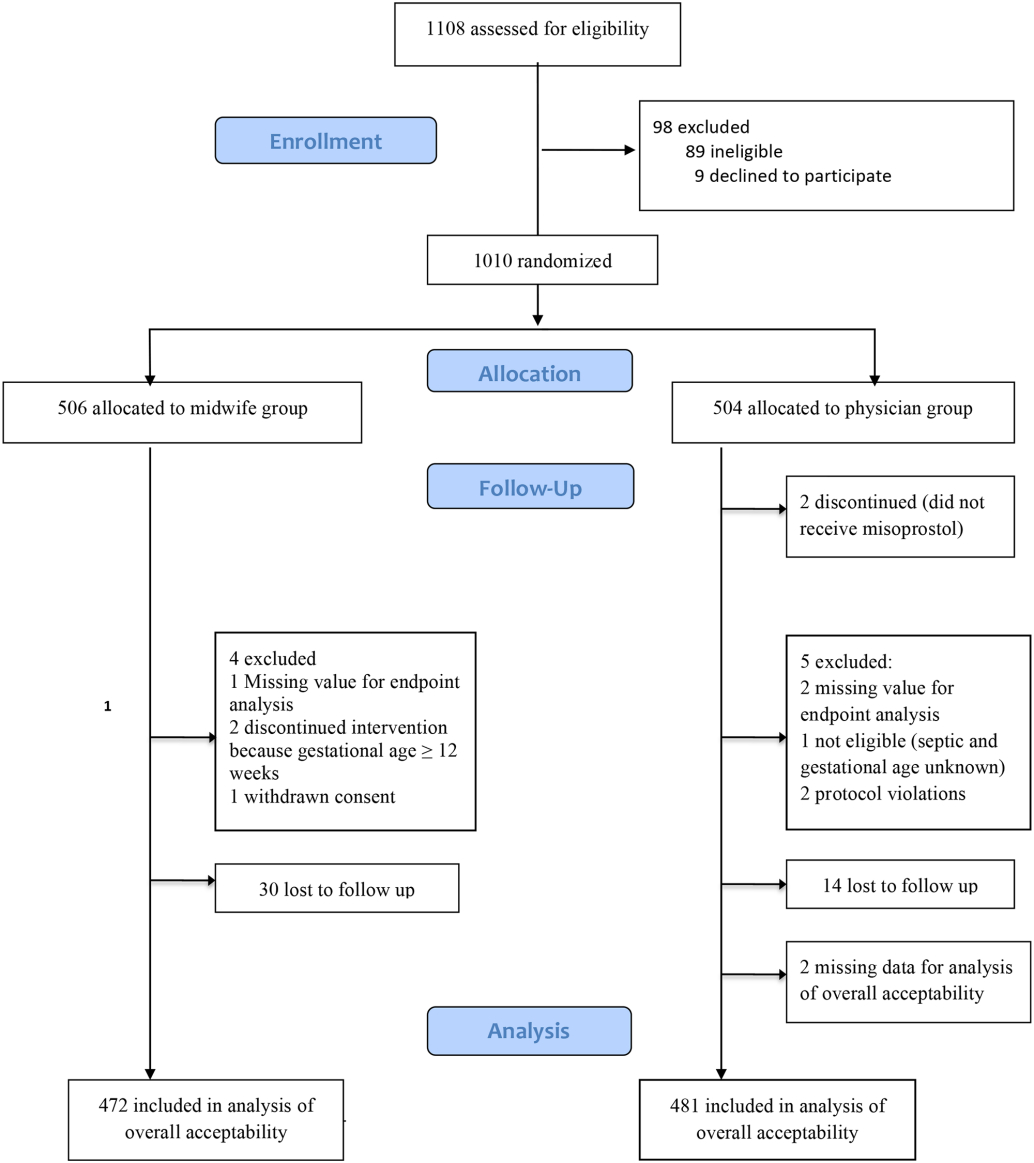
Flowchart.

In total, 1108 women were screened for eligibility and 1010 women were randomized. 955 women were successfully followed up, 472 women in the midwife group and 483 women in the physician group. The total loss to follow up was 30 in the midwife group and 14 in the physician group. In the physician group, two participants had missing values for overall acceptability analysis. The exact flow of participants can be seen in Fig 1. The loss to follow up analysis showed no difference in socio-demographic or reproductive background with the exception that women who were lost to follow up were significantly more likely to have an estimated lower gestational age (S1 Table).

### Participating providers

In total, 29 midwives and 13 physicians participated as providers in the study. Midwives and physicians were of similar age (mean 41.0 years) and had similar length of professional experience (mean 15.2 years). Physicians had longer clinical PAC experience than midwives before the study commenced (mean 8.3 years vs. 3.7 years respectively) (S2 Table).

### Socio-demographic background and reproductive history

Women´s socio-demographic background and reproductive history is presented in a previous publication [10]. Mean age of participating women was 26 years. The majority had no formal education or had completed primary school and three quarters were married or cohabiting. Mean number of pregnancies among the participants was 3.4 and about one fourth of the participants were nulliparous. One hundred and twenty-one women (12%) reported to have induced the current abortion. Mean gestational age based on clinical exam was 8 weeks and 6 days. There were no major differences in socio-demographic background or reproductive history between groups except that significantly more women in the midwife group reported to have induced the current abortion (14.6% vs. 9.7%, p-value <0.05) (data not shown).

### Women’s acceptability compared between study groups

In total 914 women (96%) reported to have experienced the treatment as easier than expected or as expected, and 942 women (99%) would recommend the treatment to a friend. With regards to overall acceptability, 49 (5%) women found the treatment not satisfactory and a total of 904 women (95%) found the treatment satisfactory (Table 1). The model-based risk difference in overall acceptability between the two study groups was 0.5% (-1.93% to 3.10%), falling within the pre-defined equivalence range of +4% to -4%. Overall acceptability, measured in expectations and satisfaction, did thereby not differ between the two study groups, midwives and physicians (Table 2).

**Table 1:** Women´s acceptability of misoprostol treatment for incomplete abortion by provider.

	Midwife n = 472 (%)	Physician n = 482 (%)	Total n = 954 (%)
**How did you perceive the treatment procedure?** (n = 954)			
As expected	104 (22.0)	104 (21.6)	208 (21.8)
Easier than expected	349 (74.0)	357 (74.0)	706 (74.0)
Worse than expected	19 (4.0)	21 (4.4)	40 (4.2)
**Would you recommend the treatment to a friend?** (n = 954)			
Yes	465 (98.5)	477 (99.0)	942 (98.7)
No	7 (1.5)	5 (1.0)	12 (1.3)
Overall acceptability* (n = 953)			
Satisfactory	449 (95.1)	455 (94.6)	904 (94.9)
Not satisfactory	23 (4.9)	26 (5.4)	49 (5.1)

* Calculated through merging of 1^st^ and 2nd question. Satisfactory = “As expected”, or “Easier than expected” and that one would recommend the treatment to a friend.

**Table 2:** Outcome of women´s overall acceptability by provider.

Acceptability	Midwife	Physician	Risk difference (95% CI)	Adjusted difference (95% CI)*
Randomised and received intervention	502	497	-	-
Successfully followed up	472	481	-	-
Overall acceptability	449 (95.1%)	455 (94.6%)	0.50% (-1.93% to 3.10%)	0.70% (-1.44% to 3.01%)

*Adjusted for age (<25 *vs* ≥25 years), marital status (single *vs* married or cohabiting), education (none or primary *vs* secondary or tertiary), number of pregnancies (1 *vs* >1), parity (para 0 *vs* ≥1) and induced current abortion (yes vs. no)

Data are n (%) unless otherwise stated

### Women´s experience of treatment and overall acceptability

Mean number of days bleeding was 5.2 days among all participants and mean reported VAS score was 3.6. Less than one fifth (16%) reported bleeding heavier than a normal menstrual bleeding following treatment and 48 women (5%) had an unscheduled visit. For 917 women (96%) the treatment was successful and did not require any further surgical intervention. In total, 32 women (3%) reported that they did not feel calm and safe following treatment (Table 3).

**Table 3:** Women’s treatment experience by overall acceptability.^a^

	Satisfactory n = 904 (%)	Not satisfactory n = 49 (%)	Total n = 953 (%)
**Number of days bleeding** (n = 950)			
Mean (SD)	5.2 (2.8)	4.7 (3.4)	5.2 (2.8)
Range	1–16	1–13	1–16
**Bleeding since treatment** (n = 951)			
Less than/same as normal menstrual bleeding	775 (85.9)	22 (44.9)	797 (83.8)
Heavier than normal menstrual bleeding	127 (14.1)	27 (55.1)	154 (16.2)
**Pain following treatment using VAS 0–10** (n = 950)			
Mean (SD)	3.5 (1.7)	4.2 (1.6)	3.6 (1.7)
Range	0–10	0–10	0–10
**Felt calm and safe following treatment** (n = 953)			
Yes	893 (98.8)	28 (57.1)	921 (96.7)
No	11 (1.2)	21 (42.9)	32 (3.3)
**Unscheduled visit**^b^ (n = 953)			
Yes	25 (2.8)	23 (47.0)	48 (5.0)
No	879 (97.2)	26 (53.0)	905 (95.0)
**Complete abortion** (n = 953)			
Yes	891 (95.8)	26 (53.1)	917 (96.2)
No	13 (4.2)	23 (46.9)	36 (3.8)
**Pelvic infection at follow up** (n = 953)			
Yes	22 (2.4)	6 (12.2)	28 (2.9)
No	882 (97.6)	43 (87.8)	925 (97.1)

^a^Calculated through merging of 1^st^ and 2nd acceptability question. Satisfactory = “As expected”, or “Easier than expected” and that one would recommend the treatment to a friend.

^b^Vaginal bleeding and/or abdominal pain.

Of the 49 (5%) women who found the treatment not satisfactory, 23 had an unscheduled visit (47%) and 27 women (55%) had experienced heavy bleeding. A total of 21 women (43%) who found the treatment not satisfactory reported not feeling calm and safe following treatment and for 23 (47%) women the treatment had failed, requiring surgical intervention. Of the 28 women had a pelvic infection at follow up, 6 (2.9%) women found the treatment non satisfactory (Table 3). Vomiting, nausea and diahorrea were the most commonly reported side effects among all women. Of those who found the treatment not satisfactory, 24 (51%) had experienced severe abdominal pain lasting >24 hours and three quarters had experienced nausea (S3 Table).

### Factors influencing women´s overall acceptability of treatment

Failed treatment (Adjusted OR 0.11 CI 0.02–0.51), not feeling calm and safe (Adjusted OR 0.07 CI 0.02–0.24), experiencing heavy bleeding (Adjusted OR 0.40 CI 0.17–0.94) and experiencing severe abdominal pain lasting >24 hours following treatment (Adjusted OR 13.62 CI 5.67–32.69), were significantly associated with non-satisfaction. The association between overall acceptability and the independent variables induced current abortion, unscheduled visit, and pelvic infection at follow up, were significant in a univariate logistic regression analysis but non-significant when added in the multivariate logistic regression model. Other background characteristics and measures related to treatment experience were not associated with overall acceptability (Table 4).

**Table 4:** Association of reproductive history and treatment experience with overall acceptability^a^ (n = 951) (Satisfactory = 1 Not satisfactory = 0).

Item	Crude OR^b^ (95% CI)	P-value	Adjusted OR (95%CI)	P-value
**Induced current abortion**				
Yes	1		1	
No	2.57 (1.30–5.10)	0.00	0.92 (0.30–2.78)	0.88
**Complete abortion**				
Yes	1		1	
No	0.01 (0.00–0.03)	0.00	0.11 (0.02–0.51)	0.00
**Unscheduled visit**				
Yes	1		1	
No	31.10 (15.63–61.86)	0.00	3.16 (0.82–12.23)	0.09
**Bleeding amount following treatment**				
Less than/same as normal menstrual bleeding	1		1	
Heavier than normal menstrual bleeding	0.13 (0.07–0.24)	0.00	0.40 (0.17–0.94)	0.03
**Felt calm and safe following treatment**				
Yes	1		1	
No	0.16 (0.00–0.03)	0.00	0.07 (0.02–0.24)	0.00
**Severe abdominal pain for >24h, following treatment**				
Yes	1		1	
No	23.68 (12.39–45.25)	0.00	13.62 (5.67–32.69)	0.00
**Pelvic infection at follow up**				
Yes	1		1	
No	5.59 (2.15–14.51)	0.00	1.06 (0.23–4.73)	0.93

^a^Calculated through merging of 1^st^ and 2nd acceptability question. Satisfactory = “As expected”, or “Easier than expected” and that one would recommend the treatment to a friend.

^b^Odds ratio (OR).

## Discussion

### Main findings

This is the first randomized controlled trial conducted, assessing women’s acceptability of being diagnosed and treated with misoprostol for incomplete abortion by midwives compared with physicians. Our study shows that misoprostol treatment of incomplete abortion by midwives compared with physicians was equally and highly acceptable to women, as have been shown in previous descriptive studies from similar contexts [18, 26]. In low resource settings where health care providers are scarce, especially in rural areas, a task shift between midwives and physicians offers a pragmatic solution, increasing access to care [27]. Published results from this randomized controlled trial show that midwives can diagnose and treat incomplete abortion with misoprostol as safely and effectively compared with physicians [10]. The high level of satisfaction among women in this study supports a task shift within PAC and the scaling up of misoprostol use for treatment of incomplete abortion at district level in Uganda. Results from this trial provided evidence to the recently published guidelines by the WHO on the health care workers role in safe abortion care [28].

Previous studies from sub-Saharan Africa have shown that a majority of women prefer misoprostol to manual vacuum aspiration for treatment of incomplete abortion, and experience side effects of misoprostol as tolerable or easily tolerable [11, 19]. Our study showed that acceptability was not determined by socio-demographic or reproductive history but rather by the treatment experience (experienced pain and bleeding, feeling calm and safe following treatment) and the treatment outcome itself, in line with previous research regarding acceptability of medical abortion from both high and low resource settings [29–31]. To our knowledge no previous study has assessed reasons for non-satisfaction regarding misoprostol treatment of incomplete abortion. However, a study from rural India assessing women´s acceptability of simplified medical abortion showed that satisfaction was affected by treatment outcome, place of follow up, unscheduled contact with the clinic, and experiencing bleeding or severe abdominal pain [31]. Furthermore, a randomized controlled trial from Sweden showed that feeling calm and safe following home administration of misoprostol, affected the abortion experience positively [30]. Patient satisfaction is an individual subjective perception closely tied to expectations on care provision and services. Counselling should focus on generating realistic expectations and ensuring that women feel calm and safe following treatment. Women´s level of satisfaction was high in our study, however our findings regarding non-satisfaction highlight the importance of counselling in connection with treatment, yielding realistic expectations, and providing detailed information regarding expected side effects, pain management, and care seeking.

We speculate that, because of fear of stigma and legal repercussions when seeking PAC, some women in this study did not disclose the fact that the abortion complications they were experiencing where due to an unsafe abortion. More women reported having induced the current abortion in the midwife group compared with the physician group, indicating that women may be more prone to share experiences of unsafe abortions with midwives. This strengthens the notion that midwives are, if trained properly, suitable to shoulder a larger responsibility in PAC.

Optimizing the role of midwives has the potential to reduce global maternal mortality and morbidity by delivering more cost-effective and accessible care. In Uganda, the scarcity of physicians is especially noticeable in rural areas where maternal mortality and morbidity is highest [9]. In response, task shifting is currently taking place at district level in Uganda but is held back by unavailability of misoprostol and lack of clear guidelines and PAC training among health care providers [8]. The Uganda Ministry of Health recently published new standards and guidelines on reducing maternal mortality and morbidity due to unsafe abortion. These guidelines support a task shift in PAC and the use of misoprostol for treatment of incomplete abortion [32]. In order to scale up misoprostol use within PAC, efforts to increase availability of misoprostol at district level need to be made. Furthermore, treatment and task shifting guidelines need to be available and made aware of among health care providers involved in PAC. Enabling midwives to play a larger part in abortion care requires that in-service PAC training and refresher courses are obtainable at district level. In addition, PAC should be incorporated into midwifery training programs, to ensure a future workforce with essential PAC skills and knowledge.

### Strengths and limitations

The design of the study, the low rate of loss to follow up, and large sample size, are strengths of the study. Because of the study design, blinding of providers or participants was not feasible. In order to reduce the risk of reporting bias, participating providers were informed to assign a different provider, for the follow up assessment, from the one who saw the woman at the first visit. However, because of the general lack of health care providers at the facilities there is a risk that some women encountered the same provider during treatment and study follow up. Another limitation is the fact that the loss to follow up was larger in the midwife group compared with the physician group. One reason could be a difference in individual providers’ ability to convey the importance of the follow up visit. This was a multi centre trial and there could be differences in women´s acceptability between health care facilities, which could be seen as a limitation. However, possible differences were accounted for in the analysis, which showed no difference in women´s acceptability between providers. This study does not attempt to attain a deeper understanding of women’s PAC experience. However, our findings provide important evidence regarding women’s acceptability of PAC and influencing factors. Further research should focus on exploring barriers to and facilitators of acceptability within PAC, using qualitative or a mixed-methods approach.

## Conclusion

Our study shows that diagnosis and treatment of incomplete abortion by midwives compared with physicians, was equally and highly acceptable to women. Our results could be of use in other contexts where access to PAC is limited due to shortages of physicians. Optimising the midwifery role and enabling task shifting within PAC should be prioritized as it may increase access to highly acceptable, safe and effective care.

## Supporting Information

S1 Table Comparison between women lost to follow up (n = 44) and women with reported primary outcome (complete/incomplete abortion) (n = 955). (DOCX)

S2 Table Background characteristics of participating PAC providers. (DOCX)

S3 Table Reported side effects among women treated with misoprostol for incomplete abortion, by overall acceptability. (DOCX)
