# Supplementary material for: Women's Acceptability of Misoprostol Treatment for Incomplete Abortion by Midwives and Physicians - Secondary Outcome Analysis from a Randomized Controlled Equivalence Trial at District Level in Uganda
Source: PLoS One. 2016 Feb 12;11(2):e0149172. doi: 10.1371/journal.pone.0149172 (PMC4752492; doi:10.1371/journal.pone.0149172)
Supplement: S1 Table — (DOCX) [file pone.0149172.s001.docx]

**S1 Table.** Comparison between women lost to follow up (n=44) and women with reported primary outcome (complete/incomplete abortion) (n= 955)

|  | Lost to follow up  n=44 (%) | Not lost to follow up  n=955 (%) | Total  n=999 (%) | **Chi2** |
| --- | --- | --- | --- | --- |
| **Age** (n=995) |  |  |  | 0.741 |
| Mean (SD) | 25.4 (6.4) | 26.5 (6.5) | 26.3 (6.5) |  |
| 16-19 | 7 (16.0) | 138 (14.5) | 145 (14.6) |  |
| 20-24 | 17 (38.6) | 307 (32.3) | 324 (32.5) |  |
| 25-34 | 14 (31.8) | 378 (39.7) | 392 (39.4) |  |
| 35-49 | 6 (13.6) | 128 (13.5) | 134 (13.5) |  |
| **Education** |  |  |  | 0.810 |
| None | 4 (9.1) | 78 (8.2) | 82 (8.2) |  |
| Primary school | 21 (47.7) | 459 (48.0) | 480 (48.1) |  |
| Secondary school | 17 (38.6) | 337 (35.3) | 354 (35.4) |  |
| Tertiary school | 2 (4.6) | 81 (8.5) | 83 (8.3) |  |
| **Marital status** |  |  |  | 0.546 |
| Married/Cohabiting | 31 (70.4) | 728 (76.2) | 759 (76.0) |  |
| Single | 11 (25.0) | 204 (21.4) | 215 (21.5) |  |
| Divorced/ Widow | 2 (4.6) | 23 (2.4) | 25 (2.5) |  |
| **Occupation** (n=996) |  |  |  |  |
| Unemployed | 22 (50.0) | 476 (50.0) | 498 (50.0) | 0.944 |
| Formal employment | 5 (11.4) | 111 (11.7) | 116 (11.6) |  |
| Self employed | 15 (34.1) | 337 (35.4) | 352 (35.2) |  |
| Student | 2 (4.5) | 28 (2.9) | 30 (3.0) |  |
| **Religion (**n=998) |  |  |  |  |
| Catholic | 22 (50.0) | 423 (44.4) | 445 (44.6) | 0.737 |
| Protestant | 14 (31.8) | 292 (30.6) | 306 (30.7) |  |
| Muslim | 6 (13.6) | 194 (20.3) | 200 (20.0) |  |
| Born again | 2 (4.6) | 45 (4.7) | 47 (4.7) |  |
| **Gestational age based on clinical exam (weeks)** (n=993) |  |  |  | 0.000 |
| Mean (SD) | 7.3 (2.1) | 8.8 (2.2) | 8.8 (2.2) |  |
| Range | 4-12 | 1-12 | 1-12 |  |
| **Number of pregnancies (**n=998) |  |  |  | 0.658 |
| Mean (SD) | 3.2 (2.3) | 3.4 (2.3) | 3.4 (2.3) |  |
| 1 | 12 (27.9) | 208 (21.8) | 220 (22.1) |  |
| 2-5 | 26 (60.5) | 588 (61.6) | 614 (61.5) |  |
| 6-20 | 5 (11.6) | 159 (16.6) | 164 (16.4) |  |
| **Number of deliveries (**n=998) |  |  |  | 0.474 |
| Mean (SD) | 2.1 (1.9) | 2.2 (2.1) | 2.2 (2.1) |  |
| 0 | 13 (30.2) | 228 (23.9) | 241 (24.1) |  |
| 1-2 | 13 (30.2) | 369 (38.6) | 382 (38.3) |  |
| 3-13 | 17 (39.6) | 358 (37.5) | 375 (37.6) |  |
| **Number of miscarriages (**n=998) |  |  |  | 0.622 |
| Mean (SD) | 1.1 (0.7) | 1.1 (0.7) | 1.49 (0.9) |  |
| 0 | 6 (14.0) | 97 (10.2) | 103 (10.3) |  |
| 1-2 | 35 (81.4) | 827 (86.6) | 862 (86.4) |  |
| 3-10 | 2 (4.6) | 31 (3.2) | 33 (3.3) |  |
