# Supplementary material for: Women's Acceptability of Misoprostol Treatment for Incomplete Abortion by Midwives and Physicians - Secondary Outcome Analysis from a Randomized Controlled Equivalence Trial at District Level in Uganda
Source: PLoS One. 2016 Feb 12;11(2):e0149172. doi: 10.1371/journal.pone.0149172 (PMC4752492; doi:10.1371/journal.pone.0149172)
Supplement: S2 Table — (DOCX) [file pone.0149172.s002.docx]

**S2 Table.** Background characteristics of participating PAC providers

|  | Midwife  n=29 (%) | Physician  n=13 (%) | Total  n=42 (%) |
| --- | --- | --- | --- |
| **Age** |  |  |  |
| Mean (SD) | 40.5 (8.2) | 42.1 (10.9) | 41.0 (9.0) |
| Median (IQR) | 41 (35-45) | 44 (31-52) | 42 (34-46) |
| **Sex** |  |  |  |
| Female | 29 (100) | 1 (7.7) | 30 (71.4) |
| Male | 0 | 12 (92.3) | 12 (28.6) |
| **Years of professional practice** |  |  |  |
| Mean (SD) | 15.4 (7.8) | 14.8 (9.9) | 15.2 (8.4) |
| Range | 2-30 | 2.5-30 | 2-30 |
| **Clinical experience in PAC before study start (yrs)** |  |  |  |
| Mean (SD) | 3.7 (3.7) | 8.3 (7.3) | 5.1 (5.4) |
| Range | 0-13.5 | 0.5-28.5 | 0-28.5 |
