# Supplementary material for: Women's Acceptability of Misoprostol Treatment for Incomplete Abortion by Midwives and Physicians - Secondary Outcome Analysis from a Randomized Controlled Equivalence Trial at District Level in Uganda
Source: PLoS One. 2016 Feb 12;11(2):e0149172. doi: 10.1371/journal.pone.0149172 (PMC4752492; doi:10.1371/journal.pone.0149172)
Supplement: S3 Table — (DOCX) [file pone.0149172.s003.docx]

**S3 Table.** Reported side effects among women treated with misoprostol for incomplete abortion, by overall acceptability*

| **Reported side effect** | **Satisfactory**  n=904 (%) | **Not satisfactory**  n=49 (%) | **Total**  n=953 (%) |  |
| --- | --- | --- | --- | --- |
| **Chill lasting >24h (n=951)** |  |  |  |  |
| Yes | 16 (1.8) | 4 (8.6) | 20 (2.1) |  |
| No | 886 (98.2) | 45 (91.8) | 931 (97.9) |  |
| **Foul smelling vaginal discharge (n=951)** |  |  |  | |
| Yes | 13 (1.4) | 1 (2.0) | 14 (1.5) | |
| No | 889 (98.6) | 48 (98.0) | 937 (98.5) | |
| **Severe abdominal pain lasting > 24h (n=951)** |  |  |  | |
| Yes | 38 (4.2) | 25 (51.0) | 63 (6.6) | |
| No | 864 (95.8) | 24 (49.0) | 888 (93.4) | |
| **Nausea (n=951)** |  |  |  | |
| Yes | 409 (45.3) | 38 (77.5) | 447 (47.0) | |
| No | 493 (54.7) | 11 (22.5) | 504 (53.0) | |
| **Vomiting (n=951)** |  |  |  | |
| Yes | 195 (21.6) | 16 (32.7) | 211 (22.2) | |
| No | 707 (78.4) | 33 (67.3) | 740 (77.8) | |
| **Diarrhoea (n=949)** |  |  |  | |
| Yes | 213 (23.6) | 10 (21.0) | 223 (23.5) | |
| No | 668 (76.4) | 38 (79.0) | 726 (76.5) | |

*Calculated through merging of 1^st^ and 2nd acceptability question. Satisfactory =”As expected”, or “Easier than expected” and that one would recommend the treatment to a friend.
